# Are the preferential patterns of myocardial iron overload preserved at the CMR follow-up?

**DOI:** 10.1186/1532-429X-14-S1-P190

**Published:** 2012-02-01

**Authors:** Antonella Meloni, Vincenzo Positano, Petra Keilberg, Daniele De Marchi, Gianluca Valeri, Massimiliano Missere, Giovanni Palazzi, Roberto Trunfio, Massimo Lombardi, Alessia Pepe

**Affiliations:** 1 CMR Unit, Fondazione G.Monasterio CNR-Regione Toscana and Institute of Clinical Physiology, Pisa, Italy; 2 Dipartimento di Radiologia, Azienda Ospedaliero-Universitaria Ospedali Riuniti "Umberto I-Lancisi-Salesi", Ancona, Italy; 3 Departement of radiology, ‘‘John Paul II’’ Catholic University, Campobasso, Italy; 4 Oncoematologia Pediatrica, Policlinico di Modena, Modena, Italy; 5 Centro Microcitemico, U.O. di Pediatria e Neonatologia, Presidio Ospedaliero Locri - A.S.L. n. 9, Locri, Italy

## Background

T2* multislice multiecho cardiac magnetic resonance (CMR) allows quantification of the segmental distribution of myocardial iron overload (MIO). This study aimed to determine if a preferential pattern of MIO was preserved between two CMR scans in thalassemia major (TM) patients.

## Methods

Among the 812 TM patients with a CMR follow-up (FU) study at 18±3 months, we selected 259 patients with significant MIO at baseline (global heart T2* <26 ms). Three short-axis views of the left ventricle were acquired and analyzed using a 16-segment standardized model. The T2* value on each segment was calculated, as well as the global value. Four main circumferential regions (anterior, septal, inferior and lateral) were defined.

## Results

The selected patient population was divided into two groups: severe (N=80, global T2* < 10 ms) and mild-moderate MIO (N=179, global T2* 10-26 ms).

For each group, there was a significant improvement in the global heart as well as in regional T2* values (P<0.0001 for all the pairwise comparisons).

For the whole patient population as well as for both two groups, at basal the mean T2* value over the anterior region was significantly lower than the mean T2* values over the other regions and the mean T2* over the inferior region was significantly lower than the T2* values over the septal and the lateral regions. The same pattern was present at the FU, with a little difference for patients with mild-moderate MIO (see figure).

**Figure 1:**
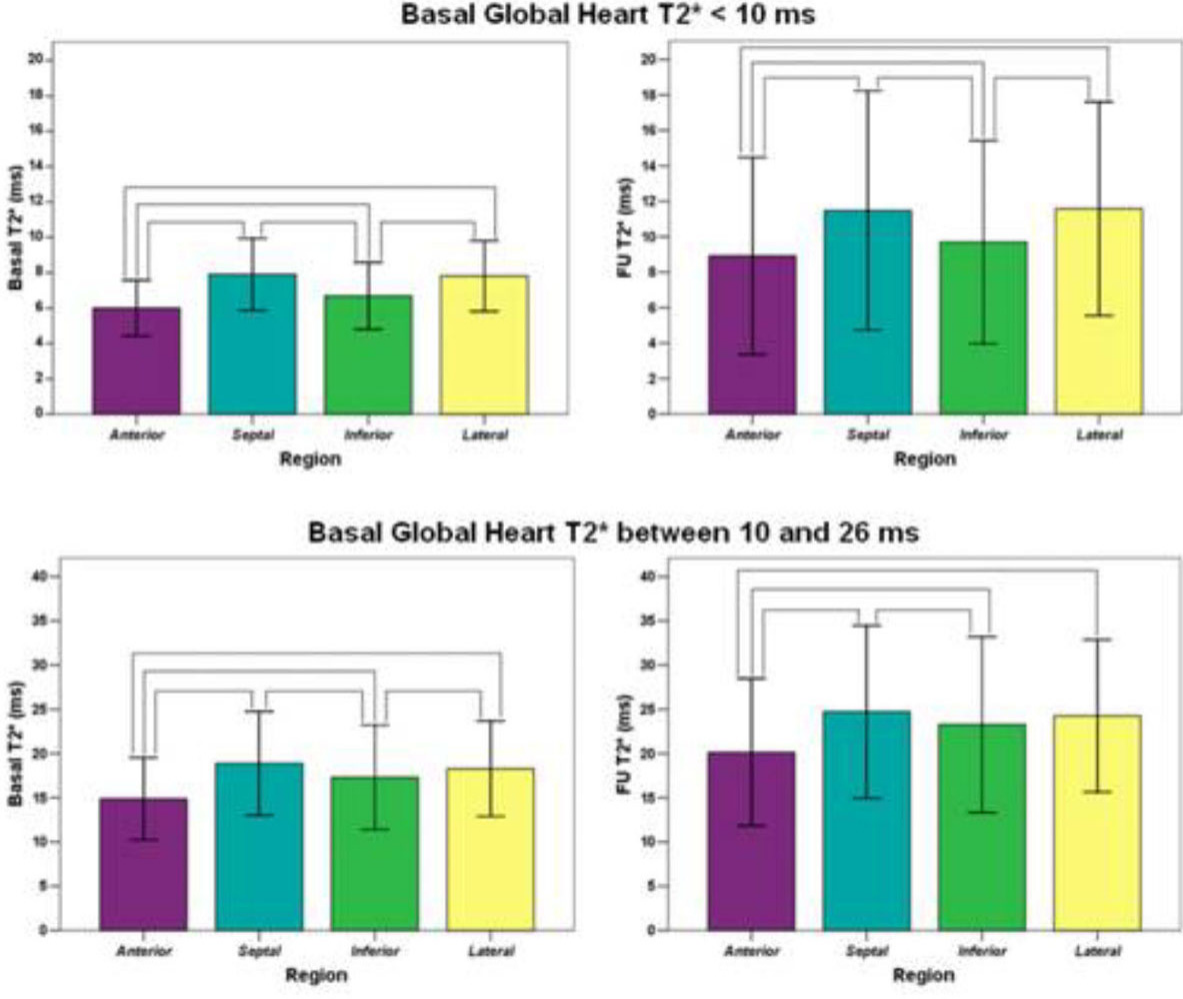


## Conclusions

A preferential pattern of iron store in anterior and inferior regions was present at both basal and FU CMRs, with an increment of T2* values at FU due to a basal CMR-guided chelation therapy. The anterior region seems to be the region in which the iron accumulates first and is removed later. Our data confirm the segmental T2* cardiac MR approach useful for identifying early iron deposit and for tailoring chelation therapy.

## Funding

“No-profit” support by industrial sponsorships (Chiesi, Apotex and GE Healtcare) and “Ministero della Salute, fondi ex art. 12 D.Lgs. 502/92 e s.m.i., ricerca sanitaria finalizzata anno 2006” e “Fondazione L. Giambrone”.

